# Transfer of Tactile Learning to Untrained Body Parts: Emerging Cortical Mechanisms

**DOI:** 10.1177/10738584241256277

**Published:** 2024-05-30

**Authors:** Sebastian M. Frank

**Affiliations:** 1 Institute for Experimental Psychology, University of Regensburg, Regensburg, Germany

**Keywords:** perceptual learning, plasticity, somatosensory cortex, somatotopy, specificity, tactile learning, transfer

## Abstract

Pioneering investigations in the mid-19th century revealed that the perception of tactile cues presented to the surface of the skin improves with training, which is referred to as *tactile learning*. Surprisingly, tactile learning also occurs for body parts and skin locations that are not physically involved in the training. For example, after training of a finger, tactile learning transfers to adjacent untrained fingers. This suggests that the transfer of tactile learning follows a somatotopic pattern and involves brain regions such as the primary somatosensory cortex (S1), in which the trained and untrained body parts and skin locations are represented close to each other. However, other results showed that transfer occurs between body parts that are not represented close to each other in S1—for example, between the hand and the foot. These and similar findings have led to the suggestion of additional cortical mechanisms to explain the transfer of tactile learning. Here, different mechanisms are reviewed, and the extent to which they can explain the transfer of tactile learning is discussed. What all of these mechanisms have in common is that they assume a representational or functional relationship between the trained and untrained body parts and skin locations. However, none of these mechanisms alone can explain the complex pattern of transfer results, and it is likely that different mechanisms interact to enable transfer, perhaps in concert with higher somatosensory and decision-making areas.

## Introduction

**Figure 1.**
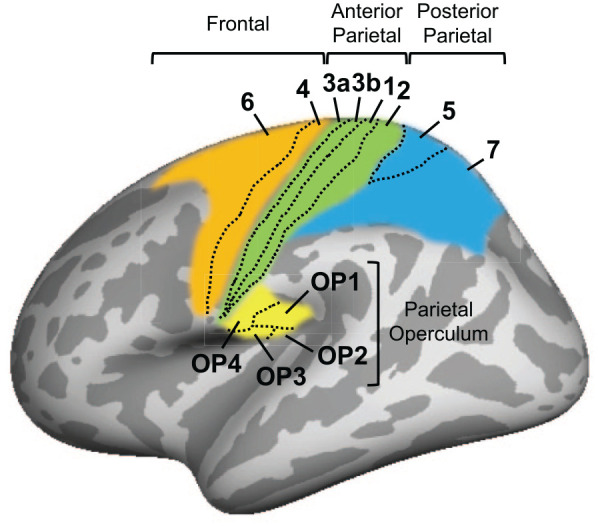
Cortical areas involved in tactile learning and transfer. Area boundaries are derived from cortical parcellations proposed by Van Essen (2005) and Glasser and others (2016). Areas are shown on the inflated left hemisphere of a template brain. Gyri are shown in light gray and sulci in dark gray. Results in monkeys suggest that area 3b should be considered the primary somatosensory cortex (S1 for short; Kaas and others 1979). Eickhoff and others (2010) proposed that somatosensory areas identified in macaque monkeys—including the secondary somatosensory cortex, the ventral somatosensory area, and the parietal ventral area (Disbrow and others 2003; Krubitzer and others 1995)—correspond to subregions of the human parietal operculum, referred to as OP1, OP3, and OP4, respectively. OP2, another subregion of the parietal operculum is part of the vestibular cortex (Eickhoff and others 2010; Frank and Greenlee 2018). Frontal and posterior parietal areas might be involved in transfer if tactile training involves learning to make decisions about tactile stimuli (Pleger and Villringer 2013; Romo and de Lafuente 2013).

**Figure 2.**
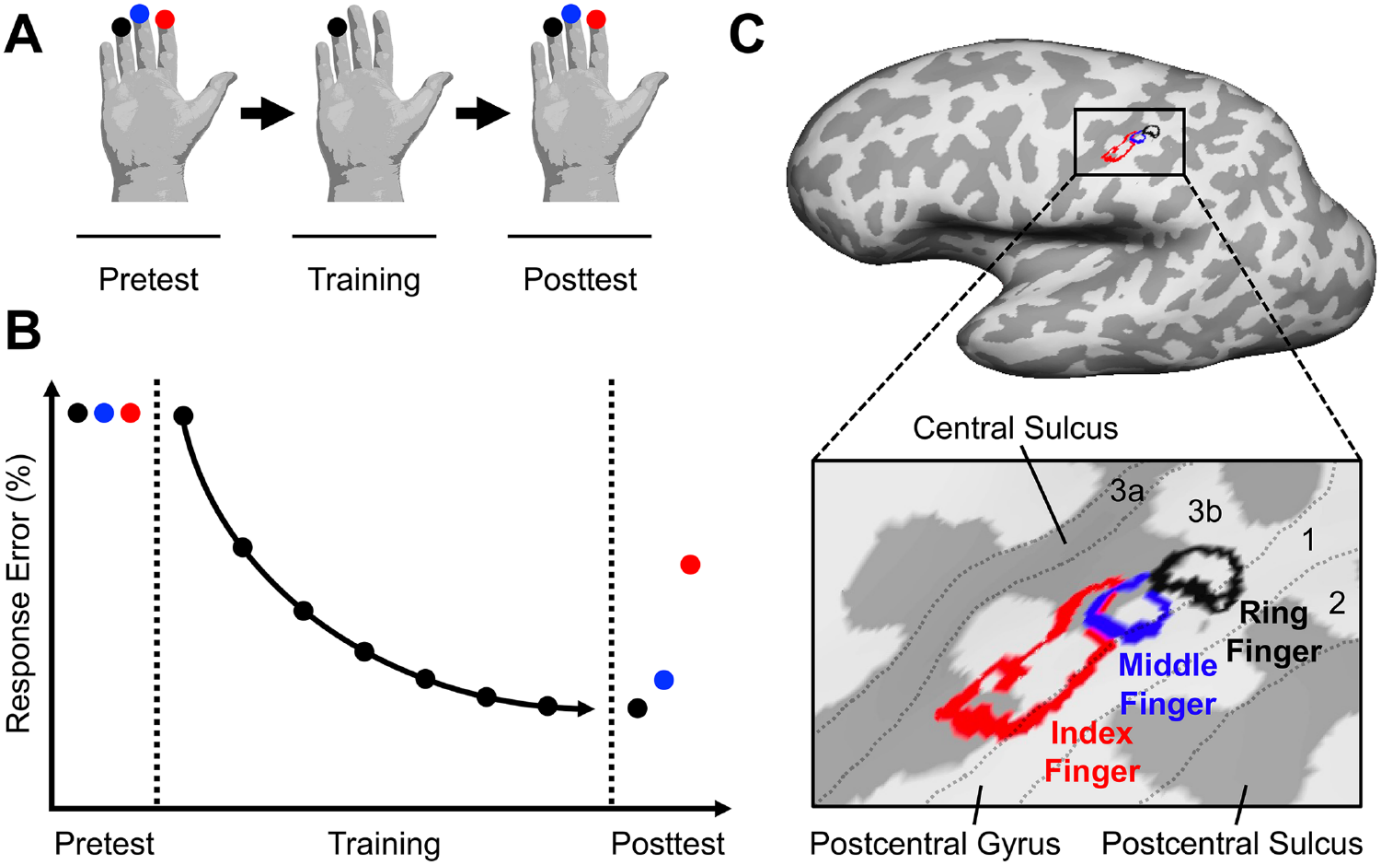
Transfer of tactile learning between trained and untrained fingers of the same hand. (A) Experimental design. The tip of the ring finger of the right hand is trained in a tactile learning task. During the pre- and posttest, performance in the tactile learning task or a related task involving the trained tactile feature is measured by using the tips of the ring finger, adjacent middle finger, and nonadjacent index finger. (B) Theoretical learning and transfer results. Pretest performance is similar among fingers, but posttest performance follows a somatotopic transfer pattern, as evidenced by few errors being made with the trained ring finger and the adjacent untrained middle finger but more errors with the nonadjacent untrained index finger. These results would suggest that tactile learning involved cortical areas with a somatotopic body map in which the middle finger is represented closer to the ring finger than the index finger. For results supporting such a behavioral pattern of transfer of tactile learning, see Harris and others (2001). (C) Cortical representations of the tips of the right ring, middle, and index fingers in a sample participant. Each fingertip was mapped with tactile stimulation during functional MRI: the representation of each fingertip was calculated by contrasting activation during stimulation of this fingertip with activation during stimulation of the other fingertips. The representations of the fingertips are shown on the participant’s inflated left hemisphere by color-coded outlines. Approximate borders among areas 3a, 3b, 1, and 2 (dashed lines) are derived from the cortical parcellation proposed by Glasser and others (2016).

Tactile perceptual learning, or *tactile learning* for short, occurs with repeated tactile experience or training and is an example of somatosensory plasticity (Buonomano and Merzenich 1998; Feldman and Brecht 2005; Seitz and Dinse 2007). A question that often arises in research on tactile learning is whether learning with one body part or skin location transfers or generalizes to untrained body parts and skin locations. This question is important because it allows conclusions to be drawn about the neuronal correlates of tactile learning (Box 1). A common finding in studies based on electrophysiology or neuroimaging in human and animal models is that tactile learning developed with repeated tactile experience or training involves somatosensory areas with topographic representations of the body surface—so-called somatotopic maps (e.g., areas 3a, 3b, 1, and 2; Figure 1; Dinse and others 2003; Elbert and others 1995; Frank and others 2022; Jenkins and others 1990; Hahamy and others 2017; Harris and others 1999; Hodzic and others 2004; Makin and others 2013; Pascual-Leone and Torres 1993; Pleger and others 2001; Pleger and others 2003; Recanzone, Merzenich, and others 1992; Wang and others 1995). In addition, numerous behavioral studies found that tactile learning developed with training transfers between trained and untrained body parts and skin locations that are represented close to each other in these somatotopic maps, such as adjacent fingers of the same hand (Dempsey-Jones and others 2016; Harrar and others 2014; Harris and others 2001; Nagarajan and others 1998; Recanzone, Jenkins, and others 1992; Sathian and Zangaladze 1997; Figure 2). This has led to the proposal of various cortical mechanisms involving areas with somatotopic maps to explain the transfer of tactile learning between trained and untrained body parts and skin locations. The aim of this review is to discuss their respective strengths (i.e., what they can explain) and weaknesses (i.e., what they cannot explain). The review begins with an introduction to pioneering experimental evidence for tactile learning and transfer published by Alfred Wilhelm Volkmann in 1858, followed by a summary of the key findings reported since then. Cortical mechanisms that have been proposed to explain the transfer of tactile learning are then presented and discussed. The review concludes with an outlook on training procedures that could facilitate transfer and with a discussion of other approaches to studying tactile learning and transfer.

**Box 1.** Specificity and Transfer of Tactile Learning.

A central question in research on tactile learning is whether learning is specific to a trained body part or skin location or transfers to untrained body parts and skin locations. For example, imagine that participants train on a tactile learning task using the right hand. In tests that are carried out before and after the end of training with the right hand (pre- and posttest, respectively), participants perform the learning task or a related task involving the trained tactile feature with the trained body part and an untrained body part (e.g., the right foot). The pretest is conducted to exclude the possibility of differences in baseline performance in the tactile learning task between the trained and untrained body parts. Note that it is possible that the pretest of the untrained body part facilitates the subsequent transfer of tactile learning to this body part; however, this possibility could be ruled out by an additional control experiment without pretesting. Theoretically, the results of the posttest with the untrained body part can show no transfer, partial transfer, or complete transfer (see Figure Box 1). In the case of no transfer, performance with the untrained body part is at baseline level, similar to pretest performance. In the case of partial transfer, performance with the untrained body part is better than in the pretest but worse than with the trained body part in the posttest. In the case of complete transfer, performance with the untrained body part is similar to that with the trained body part in the posttest. Transfer of tactile learning between trained and untrained body parts can be symmetrical, sometimes referred to as *bidirectional*, which means that transfer occurs to the same extent from trained body part A to untrained body part B and vice versa. Transfer can also be asymmetrical, meaning that transfer occurs to a greater extent from trained body part A to untrained body part B than vice versa (or the other way around). In the extreme case that there is a complete transfer from trained body part A to untrained body part B but no transfer from trained body part B to untrained body part A, this is referred to as *unidirectional transfer*. Learning specificity and transfer are important for inferring the neuronal correlates of tactile learning. High specificity—that is, no transfer of tactile learning from a trained to untrained body part—suggests that tactile learning involved neuronal processing stages with representations specific to the trained body part (e.g., area 3b). Little specificity, as shown by partial or complete transfer, suggests that tactile learning involved neuronal processing stages with overlapping or common representations of different body parts (e.g., parietal-opercular areas) or even nonsomatosensory areas (e.g., areas in the frontal or posterior parietal cortices). Figure Box 1. 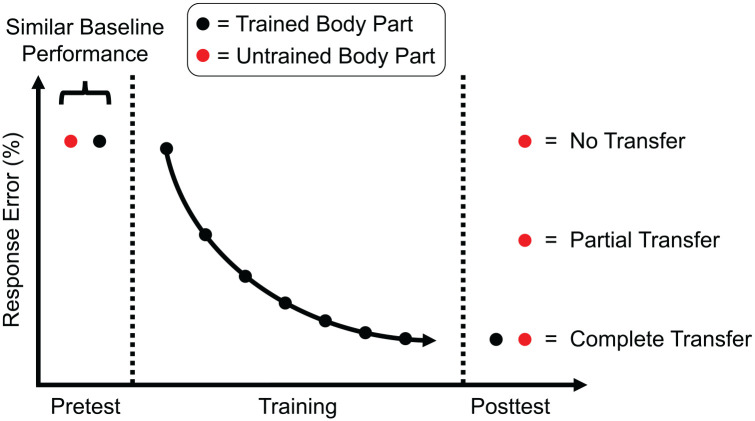 Theoretical transfer results after tactile learning. The y-axis shows performance as a percentage response error in a tactile learning task; the lower the values, the better the performance. Prior to training (pretest), performance in the tactile learning task should be similar with the trained and untrained body parts to exclude possible baseline differences in task performance between the body parts. Performance in the tactile learning task with the trained body part improves with training, indicative of tactile learning. After the end of training, transfer of tactile learning from the trained to untrained body part is examined (posttest). The results of this posttest can show no, partial, or complete transfer of tactile learning to the untrained body part.

## Historical Background

Pioneering experimental research on tactile learning and transfer was conducted by Volkmann, Gustav Theodor Fechner’s brother-in-law. In a seminal publication in 1858 entitled “Über den Einfluß der Übung auf das Erkennen räumlicher Distanzen” (“On the influence of practice on recognizing spatial distances”), Volkmann reported a series of psychophysical experiments in which he and Fechner served as participants. There were two key questions in these experiments: first, whether tactile spatial resolution improves with systematic training of a given body part or skin location; second, whether this improvement transfers to untrained body parts and skin locations. For training, Volkmann used a tactile discrimination task in which a compass circle with two points, similar to a two-point discriminator used in neurologic examinations, was briefly placed on the skin surface. Volkmann and Fechner reported whether they sensed one or two points. The smaller the receptive field of a neuron that represents a specific area of the skin, the more likely it is that participants will sense two points even though the distance between the points is very small (Mancini and others 2014; Weinstein 1968).

**Figure 3.**
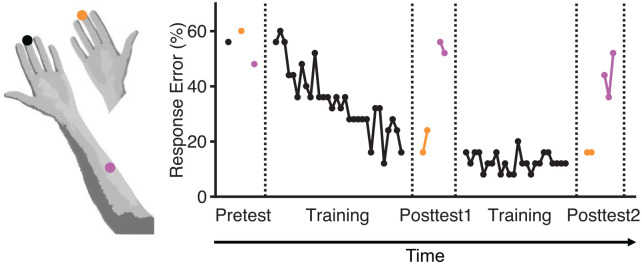
Tactile learning and transfer between trained and untrained body parts reported by Volkmann (1858; Table XV in his original publication). Volkmann placed a compass circle with two points on a given skin location and reported whether he sensed one or two points. In this experiment, Volkmann used a fixed distance between the two points of the compass circle for each body part (left middle finger, right middle finger, left forearm; tested skin locations are shown by differently colored dots in left panel) and measured how often he sensed two points in a run of 25 trials. Each dot in the right panel shows the result of a different run as a percentage response error that corresponds to the number of times that he sensed only one point in this run. The left middle finger was trained, and transfer of tactile learning to the untrained right middle finger and left forearm was examined. A pretest conducted prior to the beginning of training showed that task performance was similar with trained and untrained body parts. Transfer of learning was examined in the middle and end of training (corresponding to Posttest1 and Posttest2, respectively). The results showed that tactile learning completely transferred from the trained left middle finger to the untrained right middle finger. No transfer of tactile learning was found to the untrained left forearm.

Volkmann’s series of experiments yielded four critical results (see Tables XIII, XV, and XVI in his original publication). First, repeated training on the task improved tactile discrimination performance indicative of tactile learning. Second, tactile learning transferred between symmetrical body parts and skin locations on the left and right body sides—for example, from the trained left middle finger to the untrained right middle finger. Third, tactile learning transferred between proximal skin locations on the same side of the body—specifically, from the trained distal to untrained proximal phalanges of the same finger and between adjacent trained and untrained fingers of the same hand. Fourth, tactile learning did not transfer from the trained hand to the untrained forearm on the same side of the body. Note that Volkmann demonstrated in further experiments that tactile discrimination performance with the forearm improved with training, refuting the argument that there is no tactile learning with the forearm (see Table XIII in his original publication). A subset of Volkmann’s results is shown in Figure 3.

How can these results be explained mechanistically? Volkmann (1858) speculated that the transfer of tactile learning between symmetrical body parts and skin locations could occur via commissural connections (p. 66): “Since the symmetrically located parts of the nerve centers are demonstrably connected by a system of transverse commissures, nothing would be more obvious than to look for the transfer of practice success from one side of the body to the other in the fibers of the transverse commissures, if the indirect practice effects only occurred once and for all in symmetrically located [body] parts.” However, since his experiments showed that transfer of tactile learning occurred between nonsymmetrical body parts and skin locations, he proposed the following mechanism (p. 68): “The transferability of the influences of practice from one [body] part to another seems to depend on the proximity of their nerve sources. It is indisputable that the origins of the nerves which supply the same finger, and even those which extend to the tip of the fourth and fifth fingers, are much closer together than the origins of those fibers which supply the tip of the middle finger and the volar surface of the forearm.” Finally, he concluded (p. 68), “Rather, every nerve serving the spatial sense [as exemplified by discriminating spatial distances using the sense of touch], not in its peripheral extent, but in its central origin, represents a special organ that can be practiced, although it should be noted that the development of such a special organ [through practice] could also benefit the development of one or more others, perhaps depending on the existing neighborhoods between them.” These mechanistic explanations and conclusions initially remained speculative because they were based only on behavioral data and thus provided indirect evidence of neuronal mechanisms involved in tactile learning and transfer. Yet, later electrophysiologic studies confirmed that adjacent skin locations are represented close to each other in S1 (humans: Penfield and Rasmussen 1950; New and Old World monkeys: Kaas and others 1979), and studies in animals with sectioned corpus callosum showed that commissural connections are involved in the transfer of tactile learning between symmetrical body parts (cats: Stamm and Sperry 1957; macaque monkeys: Ebner and Myers 1962; see also the Representational Proximity and Partial Overlap in S1 section).

## Key Findings on Transfer of Tactile Learning

One could argue against the experiments by Volkmann that he and Fechner acted as experimenters and participants and therefore knew the physical distance between the two points of the compass circle before it was placed on the surface of the skin and before they were asked to report their sensations of one or two points. However, follow-up studies replicated and extended Volkmann’s original observations. The results of these studies are summarized here, sorted according to whether transfer of tactile learning was examined by using training-dependent or training-independent tactile stimulation protocols (Beste and Dinse 2013). In training-dependent tactile stimulation protocols, participants are actively trained on a tactile task, such as discriminating tactile gratings that participants explore through tactile scanning with a finger (e.g., Sathian and Zangaladze 1997) or that are pressed into a stationary finger (e.g., Wong and others 2013). In training-independent tactile stimulation protocols, participants are passively exposed to repeated tactile stimulation (e.g., on a finger) without having to perform a specific task with the tactile stimulus (e.g., Godde and others 2000).

### Training-Dependent Tactile Learning

Dresslar (1894) and Mukherjee (1933) each trained two participants on the forearm on one side of the body over several sessions using a two-point discrimination task and found that tactile learning transferred completely to the untrained forearm on the other side of the body. Follow-up studies used larger numbers of participants and examined whether tactile learning with one or more fingers of one hand transferred to untrained fingers (adjacent or nonadjacent) of the same hand as well as to fingers of the untrained hand. Learning and transfer were examined for tactile discrimination of dot patterns (Kaas and others 2013; Kauffman and others 2002; Sathian and Zangaladze 1998), orientations (Dempsey-Jones and others 2016; Harrar and others 2014; Sathian and Zangaladze 1997; Wong and others 2013), stimulation intervals (Nagarajan and others 1998), stimulation sequences (Spengler and others 1997), frequency (Harris and others 2001; Imai and others 2003), pressure (Harris and others 2001), and roughness (Harris and others 2001; Sathian and Zangaladze 1997). These studies revealed that tactile learning transferred either partially or completely from the trained finger to the adjacent untrained finger (or fingers) and to the finger symmetrical to the trained finger of the untrained hand; note, however, that Harris and others (2001) did not find transfer for frequency discrimination. Some studies also reported that tactile learning partially transferred to nonadjacent untrained fingers of the same hand (Imai and others 2003; Wong and others 2013) and nonsymmetrical fingers of the untrained hand (Imai and others 2003), but see Harris and others (2001), Harrar and others (2014), and Dempsey-Jones and others (2016), whose results showed no such transfer.

Similar transfer results were found in animal models. Results in monkeys revealed that tactile learning transferred almost completely from the trained to untrained hand in a tactile groove-smooth discrimination task (macaque monkeys; Ebner and Myers 1962) and partially from a trained finger to an adjacent untrained finger in a frequency discrimination task (owl monkeys; Recanzone, Jenkins, and others 1992). In cats, tactile learning of roughness, softness, and form discrimination transferred partially from the trained to untrained forepaw (Stamm and Sperry 1957). In rats, there was partial transfer from trained to untrained whiskers in a gap-crossing task, which decreased in magnitude with increasing representational distance between trained and untrained whiskers in S1 (Harris and Diamond 2000; Harris and others 1999).

Others studies used complex tactile stimuli for training and transfer. Arnold and Auvray (2014) found that tactile learning in a letter recognition task transferred completely among the belly, the front of the right thigh, and the right shin, regardless of which body surface was trained and which remained untrained. Another study involving training to discriminate complex tactile movement patterns by using either the palm of the hand or the sole of the foot demonstrated asymmetrical transfer of tactile learning—specifically, transfer that was greater from the trained foot to the untrained hand than vice versa (Frank and others 2022). Finally, Bach-y-Rita and others (1969) showed that blind participants learned to use a vision substitution device that converted complex visual input into vibrotactile stimulus patterns presented on the surface of the participant’s skin and that this learning transferred from one part of the body to another (e.g., from the back to the forehead or to the abdomen; Bach-y-Rita 2004).

### Training-Independent Tactile Learning

Contrary to training-dependent tactile learning, tactile learning developed with passive exposure of a finger to tactile stimulation in humans did not transfer to adjacent and nonadjacent untrained fingers of the same hand (Godde and others 2000; Muret and others 2016; Ragert and others 2008) or symmetrical fingers of the untrained hand (Dinse and others 2003; Godde and others 2000; Muret and Dinse 2018; Muret and others 2014; Muret and others 2016; Pleger and others 2001; Pleger and others 2003). There was also no transfer of tactile learning from a trained finger to the untrained forearm on the same side of the body (Muret and Dinse 2018) and from a trained toe to the untrained abdomen and metacarpal dorsal of the hand (Liang and others 2024). However, tactile learning transferred partially/completely from a trained finger to parts of the untrained face—namely, the upper lip region of the trained and untrained sides of the body and the cheek of the trained side of the body (Muret and Dinse 2018; Muret and others 2014; Muret and others 2016)—and from a trained toe to the untrained genitals (Liang and others 2024).

### Summary

Together, the results suggest that training-dependent tactile learning follows a somatotopic pattern and transfers to adjacent untrained body parts and skin locations on the same side of the body and to symmetrical body parts and skin locations on the untrained side of the body. With more complex stimuli, the generalization of tactile learning to untrained body parts and skin locations increases. In contrast to training-dependent tactile learning, transfer of training-independent tactile learning appears to follow a nonsomatotopic pattern.

## Mechanisms Involved in the Transfer of Tactile Learning

**Figure 4.**
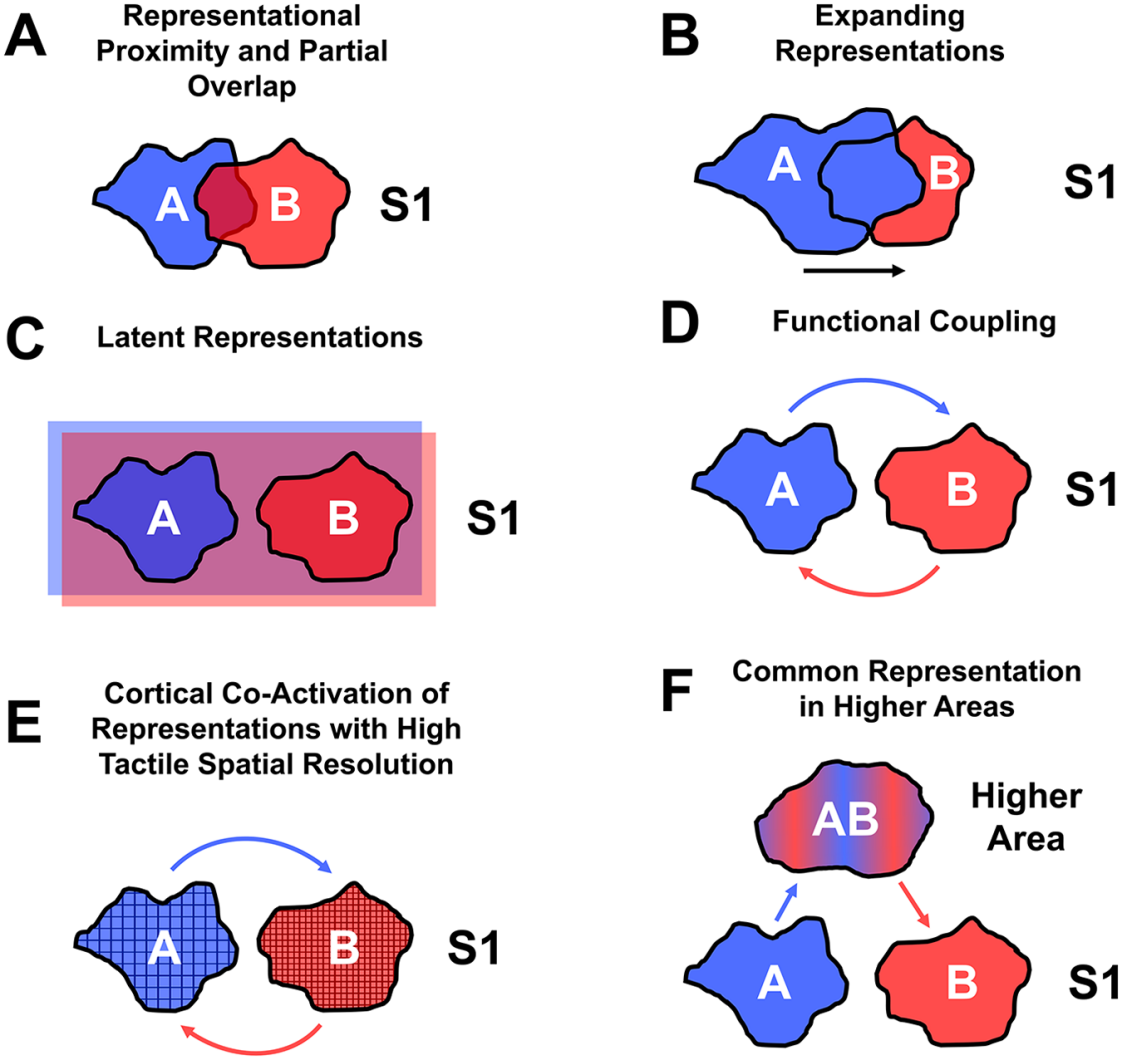
Mechanisms proposed to explain the transfer of tactile learning between trained and untrained body parts and skin locations. Blue and red blobs correspond to cortical representations of two different body parts labeled A and B. Blue is the trained body part. Red is the untrained body part. (A) Transfer due to adjacent and partially overlapping representations of trained and untrained body parts in S1. (B) Modulation of transfer with the expansion of the representation of the trained body part into the representation of the untrained body part in S1. (C) Transfer due to the latent representation of the untrained body part within the representation of the trained body part and vice versa in S1 (signified by red and blue highlighted rectangles, respectively). (D) Transfer due to functionally coupled representations of trained and untrained body parts in S1. (E) Transfer due to coactivation of the representation of the untrained body part with higher tactile spatial resolution than the trained body part in S1. (F) Transfer due to projections from S1 to higher areas with overlapping or common representations of trained and untrained body parts.

The results of training-dependent and training-independent tactile learning raise the question of which mechanisms are involved in the transfer of tactile learning and modulate the occurrence, extent, and direction of this transfer. Several mechanisms have been proposed and are discussed in turn. See Figure 4 for an illustration of the different mechanisms.

### Representational Proximity and Partial Overlap in S1

In his original work, Volkmann (1858) speculated that the transfer of tactile learning was related to the proximity of representations of trained and untrained body parts and skin locations in the central nervous system (Figure 4A). When the central representations are in proximity or adjacent or they even partially overlap, transfer of tactile learning will occur. However, when there is a large distance between the central representations, transfer will be small or absent. Volkmann’s psychophysical results—which demonstrated transfer of tactile learning to untrained skin locations of the trained finger or to adjacent untrained fingers of the same hand but no transfer to the untrained forearm on the same side of the body—support this theory because different parts of the hand are represented in proximity and have greater overlap in S1 than the representation of the forearm (humans: Penfield and Rasmussen 1950; New and Old World monkeys: Kaas and others 1979). Likewise, findings showing that the occurrence and extent of transfer decrease with greater representational distance between trained and untrained body parts in S1 support Volkmann’s idea (Harrar and others 2014; Harris and Diamond 2000; Harris and others 1999; Harris and others 2001; Imai and others 2003). His theory can also account for results in training-independent tactile learning that revealed transfer of learning to untrained body parts that are distant on the body but are represented close to each other in S1, such as transfer from the trained hand to the untrained face (Muret and Dinse 2018; Muret and others 2014; Muret and others 2016) or from the trained toe to the untrained genitals (Liang and others 2024). Yet, Volkmann’s theory reaches its limits here, as it is difficult to explain why there is no transfer of training-independent tactile learning from the trained hand to the untrained forearm, even though the face and hand are represented on opposite sides of the hand in S1 (Muret and Dinse 2018), or from a trained finger to adjacent and nonadjacent untrained fingers (Godde and others 2000; Muret and others 2016; Ragert and others 2008). Furthermore, results showing an asymmetrical transfer of training-dependent tactile learning between the hand and the foot are difficult to reconcile with his theory (Frank and others 2022).

Volkmann (1858) speculated that the transfer of tactile learning between symmetrical body parts and skin locations on left and right sides of the body—for example, between the trained finger of one hand and its symmetrical untrained finger of the other hand (Dempsey-Jones and others 2016; Harrar and others 2014; Harris and others 2001; Imai and others 2003; Kaas and others 2013; Kauffman and others 2002; Nagarajan and others 1998; Sathian and Zangaladze 1997, 1998; Spengler and others 1997; Volkmann 1858) or between the trained forearm on one side of the body and the untrained forearm on the other side of the body (Dresslar 1894; Mukherjee 1933; Volkmann 1858)—occurs through commissures connecting their central representations (i.e., the representations of these body parts and skin locations in the contralateral and ipsilateral hemispheres). Later studies in macaque and owl monkeys revealed callosal connections between somatotopic representations in the cortex, which increase in density from area 3b to areas 1 and 2, and differ in density between different body parts and skin locations within each of these areas (Killackey and others 1983). Additional callosal connections exist between higher somatosensory areas, such as the secondary somatosensory cortex (S2; macaque monkeys: Disbrow and others 2003; Jones and Powell 1969b; marmoset monkeys: Krubitzer and Kaas 1990). Results in animal models also showed that plasticity induced within the representation of a body part in S1 in one hemisphere can transfer rapidly to the corresponding representation in the other hemisphere (flying foxes and macaque monkeys: Calford and Tweedale 1990), whereas callosal lesions prevent such learning transfers between symmetrical body parts on trained and untrained sides of the body (cats: Stamm and Sperry 1957; macaque monkeys: Ebner and Myers 1962). Neurons with bilateral representations of symmetrical body parts and skin locations are found in anterior and posterior parietal regions as well as in S2 and surrounding regions (for review, see Iwamura 1998; Iwamura and others 2002). For example, Iwamura and colleagues (1994) found neurons with bilateral hand and finger representations in the intraparietal region of the postcentral gyrus (areas 2 and 5) of awake macaque monkeys and demonstrated that their ipsilateral responses depended on callosal input from the contralateral hemisphere. Neurons with bilateral representations may be crucial for the transfer of tactile learning between symmetrical skin locations on the left and right sides of the body (e.g., transfer between a trained finger of one hand to the symmetrical finger of the untrained hand).

### Expansion of Representations in S1

The transfer of tactile learning to untrained body parts and skin locations could be modulated by the expansion of representations in S1 due to repeated experience or training (Figure 4B). According to this theory, neighboring representations in S1 are in permanent competition for cortical territory. Studies in human and animal models reported that repeated stimulation of a body part with tactile input through active training or passive exposure was associated with an, at least temporary, expansion of its cortical representation and an integration of neighboring representations of body parts and skin locations (owl monkeys: Jenkins and others 1990; Recanzone, Merzenich, and others 1992; Wang and others 1995; humans: Dinse and others 2003; Hodzic and others 2004; Pleger and others 2001; Pleger and others 2003) through mechanisms such as Hebbian plasticity (Feldman and Brecht 2005). Such expansion, sometimes called *invasion* or *remapping*, into the representation of another body part may occur because this body part lacks tactile input due to, for example, disuse, deafferentation, or amputation (owl monkeys: Merzenich and others 1984; macaque monkeys: Pons and others 1991; humans: Ramachandran 1993; Lissek and others 2009). A principle underlying expansion due to missing tactile input could be that it occurs through the representations of intact body parts, which are used to compensate for the function of the missing body part in daily behavior (Hahamy and others 2017; Makin and others 2013). If the representation of a body part or skin location expands with repeated experience or training into the representation of an untrained body part or skin location, the extent of transfer of tactile learning to this untrained body part or skin location may be reduced because it has lost cortical territory (i.e., neuronal resources) to the expanding representation (Dempsey-Jones and others 2016). Theoretically, one could hypothesize that tactile skills and previously acquired tactile learning hosted within the representation of the invaded body part are transferred to the representation of the expanding body part. However, current evidence that such a novel functional representation could emerge through expansion is viewed critically (Makin and Bensmaia 2017; Makin and Krakauer 2023).

### Latent Representations in S1

**Figure 5.**
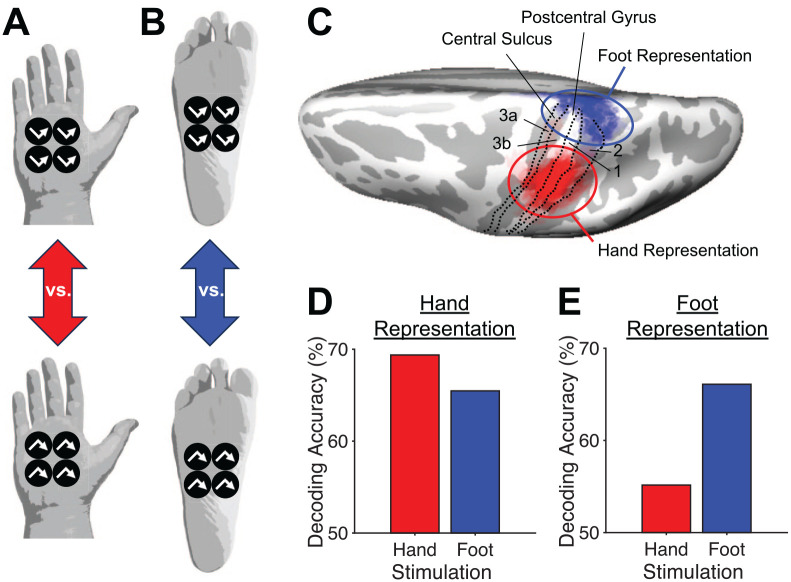
Distribution of tactile input across representations of stimulated and nonstimulated body parts in the somatosensory cortex (from Frank and others 2022). (A) Tactile stimulation conditions for the palm of the right hand. Tactile movement patterns consisting of four stimuli moving in “v”-shaped (top) and inverted “v”-shaped (bottom) trajectories from left to right were presented to the skin surface through air jets. (B) Tactile stimulation conditions for the sole of the right foot. Otherwise, same as in panel A. (C) Bird’s-eye view of the inflated left hemisphere of the same template brain as in Figure 1. Approximate boundaries among areas 3a, 3b, 1, and 2 (derived from Glasser and others 2016) are indicated by dotted lines. Red and blue colors correspond to the cortical representations of the right hand and foot, respectively, across 16 participants. Regions of greater overlap across participants are shown in more saturated colors. (D) Mean decoding accuracy of functional MRI activation patterns corresponding to “v”-shaped and inverted “v”-shaped tactile movement patterns in the cortical representation of the hand during tactile stimulation of the hand and foot across participants from panel C. Chance level of decoding accuracy corresponds to 50% on the y-axis. (E) Same as in panel D but for the cortical representation of the foot.

Despite the somatotopic organization of S1, which suggests that different body parts and skin locations are represented separately with some overlap, tactile input could be widely distributed across S1 (Muret and Makin 2021; Ramachandran 1993) through divergent thalamocortical projections to S1 (macaque monkeys: Garraghty and Sur 1990; Rausell and others 1998; for review: Jones 2000) and/or long-range corticocortical connections that cross the functional border between representations in S1 (macaque monkeys: Manger and others 1997; rats: Johnson and Frostig 2016). According to this theory, neuronal activity corresponding to tactile input would be dominant in the representation of the body part or skin location receiving the tactile stimulation in S1 and latent in other somatotopic representations (Figure 4C). This latent activity would be unmasked—possibly through downregulation of GABA (i.e., inhibitory activity; Hahamy and others 2017; Jones 2000)—if the dominant tactile input to a representation of a body part in S1 is lost, for example, due to amputation of the corresponding body part. Latent activity could support the expansion of representations of other body parts into the cortical territory of the missing body part because latent activity corresponding to tactile input from the expanding body parts was already present within the invaded representation (Hahamy and others 2017; Makin and others 2013). Recent machine learning approaches to analyzing functional MRI data in healthy human participants showed that tactile input from a given body part could be decoded in representations of body parts in S1 that do not correspond to the body part receiving the tactile stimulation, which may reflect the presence of latent activity (Frank and others 2022; Muret and others 2022). For example, tactile input from the foot could be decoded in the representation of the hand in S1 and vice versa (Figure 5). Latent activity corresponding to tactile input from a trained body part or skin location in representations of untrained body parts or skin locations in S1 during tactile training or repeated tactile exposure could be the basis for the transfer of tactile learning between these body parts and skin locations.

### Functional Coupling Between Representations in S1

Graziano and colleagues proposed that an organizing principle of the motor cortex is the co-use of different body parts in everyday behavior (Graziano 2016; Graziano and Aflalo 2007). According to this theory, in addition to a map of the body, the motor cortex is organized into functional zones that represent categories of actions—for example, hand-to-mouth or reaching-to-grasp actions. It is possible that a similar organization exists in S1 such that somatotopic representations of body parts and skin locations that are repeatedly co-used and coactivated are functionally coupled in S1, such as the hand for grasping food and the mouth for eating, or multiple fingers for tactile sensing. Tactile learning could preferentially transfer between such functionally coupled representations of trained and untrained body parts and skin locations (Figure 4D). This theory is supported by results showing transfer of tactile learning between frequently co-used body parts, such as neighboring fingers in training-dependent tactile learning (Dempsey-Jones and others 2016; Harrar and others 2014; Harris and others 2001; Imai and others 2003; Nagarajan and others 1998; Recanzone, Jenkins, and others 1992; Sathian and Zangaladze 1997; Volkmann 1858; Wong and others 2013) and the face and the hand in training-independent tactile learning (Muret and Dinse 2018; Muret and others 2014; Muret and others 2016). Furthermore, body parts such as the hand and the foot that have coevolved (Rolian and others 2010) and share anatomic similarities (e.g., similar bone structure, same number of digits) could be functionally coupled. This functional coupling could be the reason why confusion occurs between stimuli presented at the hand and foot (Badde and others 2019) and why tactile learning transfers between the hand and foot (Frank and others 2022). Functional coupling might be supported anatomically by divergent projections from the thalamus to S1 (macaque monkeys: Garraghty and Sur 1990; Rausell and others 1998; for review, Jones 2000) and/or long-range corticocortical connections between representations in S1 (macaque monkeys: Manger and others 1997; rats: Johnson and Frostig 2016). Repeated coactivation over the course of tactile training could strengthen functional coupling and result in a more integrated representation of coactivated and coupled body parts in S1 (Wang and others 1995).

### Cortical Coactivation of Representations With High Tactile Spatial Resolution in S1

This theory assumes that the transfer of tactile learning is modulated by the tactile spatial resolution of the trained and untrained body parts and skin locations. Tactile spatial resolution—measured, for example, by the two-point discrimination threshold—varies across the body surface and is highest in the face and the hands (Mancini and others 2014; Weinstein 1968). The face and hand are represented over a large area in S1, and the size of their cortical representations exceeds even what would be expected given their peripheral innervation density (Corniani and Saal 2020). If a body part or skin location with low resolution is stimulated with complex tactile cues, neurons in S1, which represent the hand and face, could support the processing of this tactile input with their high resolution. To this end, tactile input from the body part or skin location with low resolution could be shared with the representations of the hand and face in S1, leading to cortical coactivation of these representations (Figure 4E). This exchange of information between representations in S1 could occur via divergent projections from the thalamus to S1 (macaque monkeys: Garraghty and Sur 1990; Rausell and others 1998; for review, Jones 2000), by long-range corticocortical connections between representations in S1 (macaque monkeys: Manger and others 1997; rats: Johnson and Frostig 2016), and/or through connections between S1 and higher somatosensory areas, such as S2 in the parietal-opercular cortex (macaque monkeys: Disbrow and others 2003; Jones and Powell 1969a; marmoset monkeys: Krubitzer and Kaas 1990; humans: Eickhoff and others 2010). With repeated coactivation over the course of training in a tactile learning task, the cortical representations of the hand and face will learn the task; in other words, learning will transfer. The theory of cortical coactivation assumes that the representations of the hand and face are coupled and share tactile information with other somatotopic representations in S1. This has similarities to mechanisms that assume functional coupling and latent representations in S1. However, it differs from these mechanisms: first, coupling between representations does not depend on co-use; second, latent activations in the hand and face representations are functionally more relevant to tactile learning and transfer than latent activations in other somatotopic representations.

**Figure 6.**
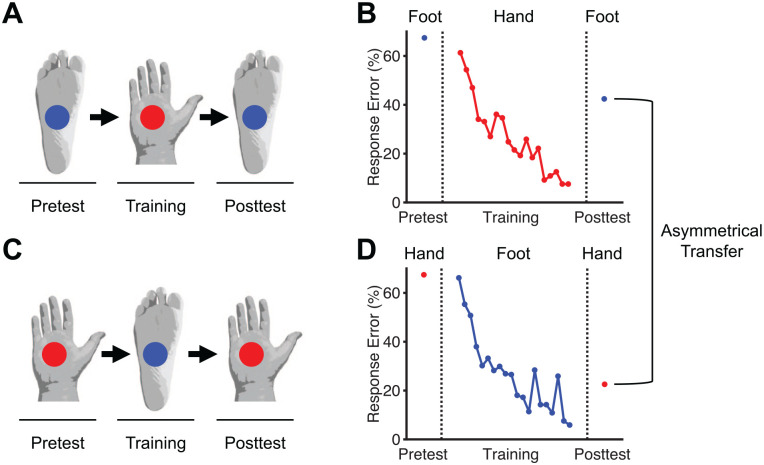
Asymmetrical transfer of tactile learning (from Frank and others 2022). (A) Design of tactile learning with the palm of the right hand and transfer test with the untrained sole of the right foot. (B) Mean learning and transfer results across six participants recruited for the study in panel A. (C) Design of tactile learning with the sole of the right foot and transfer test with the untrained palm of the right hand. (D) Mean learning and transfer results across six new participants recruited for the study in panel C.

A recent study (Frank and others 2022) investigated this theory for tactile stimulation of the hand and foot (Figures 5 and 6). The hand has higher spatial resolution to discriminate tactile cues than the foot (Mancini and others 2014; Weinstein 1968). The study found that the representation of the hand was coactivated during tactile stimulation of the foot (Figure 5D). In contrast, cortical coactivation in the opposite direction (i.e., of the cortical representation of the foot during tactile stimulation of the hand) was much less pronounced (Figure 5E). The theory of cortical coactivation predicts that tactile learning will transfer to the untrained hand due to repeated coactivation of its cortical representation over the course of training with the foot. Confirming this prediction, the study revealed that tactile learning transferred to a greater extent from the trained foot to the untrained hand than vice versa, corresponding to asymmetrical transfer (Figure 6).

### Common Representation in Higher Somatosensory Areas and Involvement of Decision-Making Areas

The transfer of tactile learning to untrained body parts could occur in higher processing stages instead of S1 (Figure 4F). The somatosensory system is hierarchically organized, and tactile information from S1 is further processed in higher areas in the parietal-opercular and anterior and posterior parietal cortex in pathways specialized for features such as texture, shape, or orientation (for review, see Sathian 2016). Neurons in these areas have receptive fields that span large portions of the skin surface (e.g., multiple fingers or the whole hand) and are often bilateral (macaque monkeys: Fitzgerald and others 2006; Krubitzer and others 1995; Robinson and Burton 1980; Taoka and others 2016; for review, see Iwamura 1998; Iwamura and others 2002). These neurons could be part of a network with a common high-level representation of different body parts based on categorical information, such as body part identity (e.g., common representation of left and right hands) and body part laterality (e.g., common representation of left hand and foot; Tamè and Longo 2023). Results in humans (Hodzic and others 2004; Pleger and others 2003) and macaque monkeys (Ridley and Ettlinger 1976) showed that higher somatosensory areas, such as S2, are involved in tactile learning. Therefore, it is possible that the representation of a trained body part or skin location in S1 projects tactile information during training to higher areas with a common representation of trained and untrained body parts and skin locations, which could result in tactile learning in each of these body parts and skin locations. Through feedback from higher areas back to S1, tactile learning would also be reflected in the representation of the untrained body part or skin location in S1 (Figure 4F).

Note that it is possible that the transfer of tactile learning in tasks requiring decision making about the tactile stimuli is modulated by or involves decision-making areas in the frontal and posterior parietal cortices (Pleger and Villringer 2013; Romo and de Lafuente 2013). Results in macaque monkeys showed that activity gradually builds up from somatosensory areas in the anterior parietal lobe, which encode tactile stimulus features, to premotor areas in the frontal lobe, which are involved in decision making about the tactile stimuli (de Lafuente and Romo 2006; Hernández and others 2002). Other areas crucial to perceptual decision making, such as the dorsolateral prefrontal cortex (humans: Pleger and others 2006) and the lateral intraparietal area in the posterior parietal cortex (macaque monkeys: Law and Gold 2008; Shadlen and Newsome 2001), may also be involved.

## Discussion of Mechanisms Involved in Transfer of Tactile Learning

Beginning with Volkmann, several mechanisms have been proposed to explain the transfer of tactile learning between trained and untrained body parts and skin locations. These mechanisms are similar in some aspects. For example, many mechanisms assume that the transfer occurs between somatotopic representations in S1 and that there is a relationship, spatial or functional, between the representations of trained and untrained body parts and skin locations in S1. It is possible that different mechanisms interact to enable the transfer of tactile learning and that the extent to which each mechanism is involved varies with the trained and untrained body parts and skin locations, the complexity of the tactile stimulus, and the tactile stimulation protocol. Representational proximity and partial overlap could be involved in transfer when trained and untrained body parts and skin locations are represented close to each other in S1, whereas mechanisms such as latent representations, functional coupling, and cortical coactivation might come into play when the representations of trained and untrained body parts and skin locations in S1 are farther apart. It is also possible that transfer observed on the level of S1 reflects feedback from higher areas with a common representation of trained and untrained body parts and skin locations. This mechanism could be involved in tactile learning tasks in which stimuli consist of complex tactile features (e.g., learning of tactile letters presented to the skin surface; Arnold and Auvray 2014), which involve processing in higher areas (Sathian 2016).

It is likely that different mechanisms are involved and interact, as each mechanism alone can explain only a limited number of findings. For example, representational proximity and partial overlap cannot easily explain transfer of tactile learning that occurs specifically to just one of two adjacent representations of untrained body parts in S1 (Muret and Dinse 2018; Muret and others 2014; Muret and others 2016), as well as asymmetrical transfer and transfer between nonadjacent representations in S1 (Frank and others 2022). Mechanisms assuming functional coupling between representations of different body parts in S1 and cortical coactivation of representations with high tactile spatial resolution are limited because corticocortical connections could be sparse (New and Old world monkeys: Chand and Jain 2015; Fang and others 2002; Wang and others 2013; humans: Glasser and others 2016). Furthermore, it is unclear whether corticocortical connections exist among all representations in S1. Recent decoding results of brain imaging data in human participants support the idea of latent representations in S1 (Frank and others 2022; Muret and others 2022), but this mechanism cannot easily account for results that show asymmetrical transfer of tactile learning between trained and untrained body parts. In addition, the extent to which tactile information from a stimulated body part is found in the cortical representation of an unstimulated body part appears to vary with respect to the stimulated and unstimulated body parts (Frank and others 2022). The results also revealed a major difference in the transfer of tactile learning from a trained finger to the symmetrical finger of the untrained hand and to adjacent fingers of the trained hand between training-dependent and training-independent tactile learning (transfer occurs in the former but not in the latter type of learning). Whether this reflects that fundamentally different mechanisms are involved in these two types of tactile learning needs to be examined in future research. Because transfer in training-independent tactile learning does not follow a strict somatotopic pattern, such transfer could more strongly involve mechanisms other than representational proximity and partial overlap (e.g., functional coupling or cortical coactivation).

Recent results showed that priors about where the limbs are usually located in space not only influence tactile perception (Badde and others 2019) but may be reflected in the neuronal organization of the tactile system (Badde and Heed 2023). Therefore, how the body is usually located in space may further modulate the transfer of tactile learning. For example, if the hands are crossed during tactile learning so that the untrained hand rests at the default position of the trained hand, the transfer of tactile learning from the trained to untrained hand could be facilitated (Badde and Heed 2023).

Future research should address the role of parietal opercular and anterior and posterior parietal areas in the transfer of tactile learning. Many characteristics of these areas, such as overlapping and bilateral representations of body parts, could support the transfer of tactile learning. It is also possible that subcortical regions in the thalamus and brainstem are involved in transfer. Somatotopic representations in the thalamus have a high degree of interaction with corresponding representations in S1 to maintain a match between subcortical and cortical representations (Zembrzycki and others 2013), and changes in somatotopic maps after loss of tactile input occur subcortically in a manner like that found in S1 (Jain and others 2008; Jones and Pons 1998).

## Facilitation of Learning Transfer

Although the specificity of tactile learning for a trained body part or skin location has theoretical significance, for practical application (e.g., for rehabilitation after peripheral or central damage to the somatosensory system) it would be helpful if there was a pronounced transfer of tactile learning. Research in visual perceptual learning, or *visual learning* for short—which refers to a performance improvement in a visual task through repeated experience or training (Frank and others 2021; Seitz and Dinse 2007)—found various ways to facilitate learning transfer. Similar to tactile learning, visual learning can be specific to a trained retinal location (e.g., Karni and Sagi 1991). However, specific training procedures—such as performing an irrelevant task or a brief pretest in the training task at untrained retinal locations (Xiao and others 2008; Zhang and others 2010) or training with varied stimuli to reduce visual adaptation (Harris and others 2012)—facilitate the transfer of visual learning to untrained retinal locations. The difficulty of the training task also plays a role, as prolonged training at a perceptual threshold (i.e., difficult training) promotes specificity whereas easier training sets promote transfer (Ahissar and Hochstein 1997; Hung and Seitz 2014). Furthermore, longer training results in high specificity, whereas learning in earlier training phases is more easily transferable (Jeter and others 2010).

The extent to which these mechanisms facilitate the transfer of tactile learning requires further research. However, some of the mechanisms facilitating transfer in visual learning might not be as effective in tactile learning. For example, many studies reported that a pretest on untrained skin locations did not result in similar transfer to each untrained skin location (training-dependent tactile learning: Dempsey-Jones and others 2016; Frank and others 2022; Harrar and others 2014; Imai and others 2003; Kaas and others 2013; Volkmann 1858; Wong and others 2013; training-independent tactile learning: Dinse and others 2003; Godde and others 2000; Liang and others 2024; Muret and Dinse 2018; Muret and others 2014; Muret and others 2016; Pleger and others 2001; Pleger and others 2003; Ragert and others 2008). Furthermore, tactile learning transferred to a limited extent from trained to untrained fingers after a single training session (Harris and others 2001), whereas extensive training over many sessions resulted in pronounced transfer of learning from trained to untrained fingers (Imai and others 2003). Even with a training paradigm based on procedures that facilitate transfer in visual learning (Harris and others 2012; Zhang and others 2010), the extent of transfer of tactile learning was still limited (Harrar and others 2014).

## Other Approaches to Studying Tactile Learning and Transfer

The majority of experiments reviewed were carried out via passive touch, which involves the activation of exteroceptors in the skin surface through external tactile stimulation. However, tactile learning can also occur through active touch—that is, intended movements of a given body part to generate touch with an object (sometimes referred to as *tactile scanning*). The latter includes exteroceptive and proprioceptive inputs—specifically, exteroceptive inputs arising from contact with the object and proprioceptive inputs about the position and movement of the body part used for touching. Blindfolded participants can identify the shape of an object (e.g., a cookie-cutter star) by manually exploring its edges but without ever feeling the object’s overall shape on their skin (Gibson 1962). This suggests that manual exploration extracts object-specific features that remain invariant even when exteroceptive and proprioceptive inputs continuously change. It is not certain whether and, if so, how the transfer of tactile learning differs in learning that was developed with active versus passive touch. Similar to transfer after tactile learning with passive touch, experiments on tactile learning with active touch showed that learning transferred partially/completely between trained and untrained adjacent fingers and their symmetrical fingers of the untrained hand (Sathian and Zangaladze 1997). Yet, whether similar or different mechanisms are involved in the transfer of tactile learning in active and passive touch is unclear and should be clarified in future research. Moreover, it should be noted that there are other types of transfer, such as cross-sensory transfer, which can be modulated by learning. For example, Bach-y-Rita and others (1969) demonstrated that tactile learning of visual flow information that was converted into vibrotactile stimulus patterns could be used by blind participants for navigation. Furthermore, using the Tadoma method, deafblind participants learned to capture speech information by placing their hands on a speaking face (e.g., Reed 1996). A thorough investigation of the extent to which such tactile learning transfers to untrained body parts and skin locations could provide important insights into the mechanisms involved in cross-sensory transfer.

## Conclusion

Since Volkmann’s pioneering research in the mid-19th century, tactile learning and transfer have been studied for various body parts and skin locations in human and animal models. Overall, the results suggest that several cortical mechanisms are involved in the transfer of tactile learning. For many of these mechanisms, somatotopic representations of trained and untrained body parts and skin locations in S1 play a significant role. Future research is needed to understand how different mechanisms interact in the transfer of tactile learning and which training procedures facilitate transfer.
